# Adhésifs en prothèse amovible complète: indications, propriétés et applications cliniques

**DOI:** 10.11604/pamj.2026.53.91.45923

**Published:** 2026-02-20

**Authors:** Rabia Mekayssi, Hassnae Benyahia, Salwa Berrada, Amal Sefrioui

**Affiliations:** 1 Service de Prothèse Adjointe, Faculté de Médecine Dentaire, Université Mohammed V, Rabat, Maroc

**Keywords:** Prothèse amovible complète, rétention, adhésifs prothétiques, stabilité prothétique, xérostomie, Complete removable denture, retention, denture adhesives, stability, xerostomia

## Abstract

Lorsque les conditions anatomophysiologiques sont défavorables, la rétention et la stabilité des prothèses amovibles complètes deviennent difficiles à obtenir. Dans ces situations, les adhésifs constituent une solution complémentaire utile pour optimiser l'équilibre prothétique, en particulier lorsque les alternatives comme les attachements radiculaires ou implantaires ne sont pas envisageables. Une revue narrative de la littérature a été réalisée à partir d'articles publiés entre 1989 et 2023, concernant les propriétés, les indications et les applications cliniques des adhésifs pour prothèses complètes. Les bases consultées comprenaient PubMed, Scopus et Google Scholar. Les adhésifs améliorent la rétention et la stabilité grâce à une couche viscoélastique favorisant l'adhésion aux structures ostéo-muqueuses. Ils présentent également des avantages biologiques (répartition des forces, réduction des ulcérations, diminution de la pénétration alimentaire) et psychologiques (augmentation du confort et de la confiance). Les principales indications incluent la résorption sévère, la xérostomie, les difficultés d'adaptation à une nouvelle prothèse, les réhabilitations provisoires et les besoins fonctionnels spécifiques. Leur utilisation nécessite un protocole rigoureux incluant un nettoyage adéquat, une application maîtrisée selon la forme galénique et une hygiène bucco-prothétique stricte. Les adhésifs jouent un rôle essentiel dans l'amélioration de la rétention, de la stabilité et du confort des prothèses amovibles complètes. Leur efficacité est particulièrement marquée chez les patients présentant une résorption sévère ou une xérostomie. Toutefois, ils ne doivent pas compenser une mauvaise adaptation prothétique. Les formulations modernes offrent une meilleure sécurité, notamment grâce à l'absence de zinc dans la majorité des produits récents.

## Abstract (en)

This article explores the importance of adhesives in improving the retention and stability of complete removable dentures. These adhesives are recommended when complementary solutions prove impossible and when anatomical and physiological conditions do not allow for achieving optimal prosthetic balance. In this context, the use of adhesives provides a practical solution by ensuring sufficient retention while reducing uncomfortable movements and irritations of the oral mucosa. Adhesives are available in various galenic forms, and the choice depends on the specific needs and comfort of each patient. By facilitating initial adaptation and enhancing daily quality of life, denture adhesives play an essential role in supporting and improving the well-being of patients with complete removable dentures.

## Introduction

Lorsque les conditions anatomophysiologiques sont compromises, l'équilibre d'une prothèse amovible complète (PAC) représente un véritable défi clinique. Les solutions complémentaires de rétention telles que les attachements radiculaires ou implantaires peuvent être indiquées, mais ne sont pas toujours réalisables en raison de contraintes cliniques, financières ou anatomiques [1,2]. Dans ce contexte, les adhésifs pour prothèses complètes constituent une alternative pertinente pour optimiser la stabilité, en particulier au niveau mandibulaire, où la rétention demeure difficile [3,4].

Les adhésifs jouent un rôle essentiel pour améliorer la rétention, le confort et la qualité de vie des patients édentés. Toutefois, ils ne peuvent pas compenser des prothèses ne respectant pas les principes biomécaniques d'équilibre. Leur efficacité dépend de leur composition, de leur forme galénique et du respect des protocoles d'utilisation [5,6]. L'objectif de cet article est d'exposer les propriétés, les indications et les applications cliniques des adhésifs en prothèse amovible complète.

## Méthodes

Cette revue narrative a été conduite en consultant la littérature portant sur les adhésifs pour prothèses complètes. Les bases de données PubMed, Scopus et Google Scholar ont été interrogées pour la période 1989-2023. Les mots-clés utilisés incluaient dentures adhésives, complètes dentures, rétention, stabilité, et mastication. Les études portant sur les formulations, les effets cliniques, les avantages biomécaniques, biologiques et psychologiques, ainsi que les recommandations d'usage ont été incluses. Les articles non pertinents ou portant sur les prothèses partielles ont été exclus.

## Résultats

**Composition et formulation:** l'adhésif utilisé en prothèse amovible complète est un dispositif médical de classe I, conforme à la norme internationale NF EN ISO 10873, qui en définit les exigences et méthodes d'essai. Son objectif est de renforcer l'adhésion de la prothèse à la fibromuqueuse, améliorant ainsi la stabilité de la prothèse et favorisant une intégration biologique et fonctionnelle optimale. Les adhésifs pour prothèses sont principalement composés de trois types d'ingrédients: les matériaux responsables des propriétés adhésives, tels que la gomme de karaya, la tragacanthe, l'acacia, la pectine et la gélatine, ainsi que des polymères synthétiques comme le polyéthylène et le polyvinyl acétate; des agents antimicrobiens, tels que l'hexachlorophène, le tétraborate de sodium et l'éthanol; et enfin des additifs, agents mouillants et plastifiants, tels que l'huile de gaulthérie et l'huile de menthe poivrée. Ces composants sont essentiels pour assurer l'efficacité et la stabilité des adhésifs utilisés en prothèse amovible complète [4,6,7].

**Figure 1:**
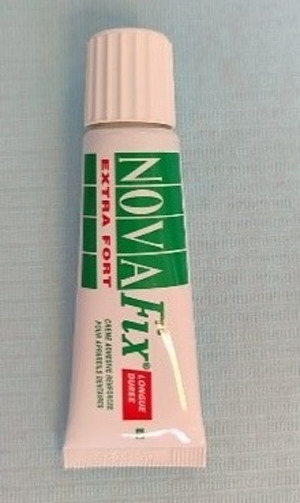
crème adhésive

Les adhésifs pour prothèses se présentent sous différentes formes galéniques afin de répondre aux besoins et préférences spécifiques des utilisateurs [7]. On les retrouve sous les formes suivantes:

***Crème ou pâte:*** c'est la forme la plus courante. Elle est facile à appliquer directement sur la prothèse, offrant une bonne répartition et une adhérence durable (Figure 1).

**Figure 2:**
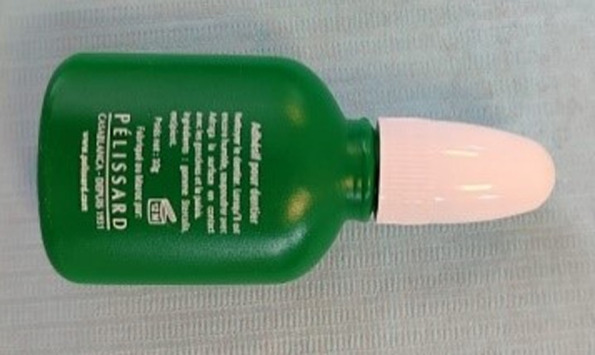
poudre adhésive

***Poudre:*** cette forme sèche se transforme en gel adhésif au contact de la salive, permettant une application uniforme et souvent plus facile à doser (Figure 2).

***Bandes ou feuilles:*** ce sont des feuilles d'adhésif pré-découpées, faciles à appliquer et à enlever. Elles constituent une solution pratique pour ceux qui préfèrent éviter l'utilisation de crèmes ou de poudres.

***Gel:*** de consistance semi-liquide, le gel combine les avantages de la crème et de la poudre, offrant une application facile ainsi qu'une bonne rétention.

### Propriétés biomécaniques, biologiques et psychologiques

**Biomécanique:** les adhésifs pour prothèses complètes présentent plusieurs avantages biomécaniques qui contribuent à améliorer la stabilité et la fonction des prothèses. Ils renforcent la stabilité et la rétention en formant une couche viscoélastique qui permet à la prothèse de mieux adhérer à ses structures d'appui. Ces propriétés biomécaniques font des adhésifs un outil intéressant pour optimiser l'équilibre de la prothèse [8-10].

**Biologique:** les adhésifs, grâce à leurs composants, favorisent la protection des structures d'appui ostéo-muqueuses. En maintenant la prothèse fermement en place et en assurant une répartition uniforme des forces masticatoires sur la surface des tissus sous-jacents, on ralentit ainsi le processus de résorption osseuse. De plus, les adhésifs agissent comme une barrière protectrice, réduisant les frottements et prévenant les ulcérations et inflammations. Ils empêchent également l'infiltration de particules alimentaires dans les hiatus qui se créent entre la prothèse et les tissus, réduisant ainsi le risque d'infections et d'inflammations. Enfin, certains adhésifs contiennent des agents hydratants qui contribuent à la lubrification des structures d'appui, largement recherchée dans le cas de sécheresse buccale [11,12].

**Psychologique:** les adhésifs augmentent le confort, la fonctionnalité des prothèses, contribuant ainsi à une meilleure qualité de vie globale pour les patients édentés, ce qui offre des avantages psychologiques indéniables [13].

### Indications

**Résorption sévère:** les adhésifs offrent une solution non invasive et efficace pour améliorer la rétention et la stabilité des prothèses amovibles complètes quand les autres solutions ne peuvent pas être exploitées [13].

**Nouvelle prothèse:** lors de la mise en place de nouvelles prothèses, les patients peuvent rencontrer des difficultés d'adaptation initiale. L'utilisation d'adhésifs peut faciliter cette période souvent redoutée par le patient non habitué à l'amovibilité de la prothèse [4,7].

**Activités spécifiques:** les adhésifs sont également recommandés pour les patients engagés dans des activités nécessitant une sécurité prothétique accrue, comme les discours en public, les repas importants ou les activités physiques modérées. La confiance accrue en la stabilité de la prothèse permet aux patients de mener une vie plus active et sociale [8,13].

**Réhabilitation provisoire et immédiate:** en attendant une réadaptation prothétique ou une intervention chirurgicale, les adhésifs peuvent fournir une solution temporaire pour améliorer la rétention et le confort des prothèses. Cela est particulièrement utile pour les patients en transition entre différentes étapes de traitement prothétique [7].

**La gériatrie:** réussir l'intégration d'une nouvelle prothèse amovible chez les seniors et les grands seniors est souvent un défi. Le recours aux adhésifs, même s'il est prévu temporairement, s'avère souvent essentiel pour améliorer la rétention et le confort. Il aide particulièrement les patients ayant des troubles de mémoire à mieux s'adapter à leurs prothèses au quotidien [4].

**Xérostomie:** les causes de la xérostomie sont multiples et incluent la radiothérapie, les changements hormonaux, les effets secondaires des médicaments et des troubles systémiques. La diminution du flux salivaire rend difficile les forces d'adhésion nécessaires à la rétention et au maintien en place des prothèses amovibles [13,14].

**Enregistrement du rapport maxillo-mandibulaire:** pour obtenir un rapport maxillo-mandibulaire précis, il est essentiel que la maquette d'occlusion soit stable. Le recours aux adhésifs pour prothèse amovible, qu'ils soient sous forme de poudre ou de crème, permet d'assurer la stabilité de la maquette d'occlusion sur les structures d'appui, facilitant ainsi les étapes de détermination et d'enregistrement du rapport maxillo-mandibulaire [7].

**Protocole clinique d'application:** l'utilisation des adhésifs nécessite le respect d'un protocole pour leur bonne exploitation. L'application des adhésifs passe par plusieurs étapes:

### Préparation de la prothèse et les structures d'appui

***Nettoyage:*** avant l'application de tout adhésif, il est essentiel de nettoyer soigneusement la prothèse et la muqueuse buccale avec une brosse douce et un nettoyant spécifique pour prothèses dentaires à base de (peroxyde de sodium) ou d'autres molécules afin d'éliminer les résidus alimentaires et la plaque bactérienne. Ce nettoyage garantit une surface propre, favorisant une meilleure adhésion.

**Figure 3:**
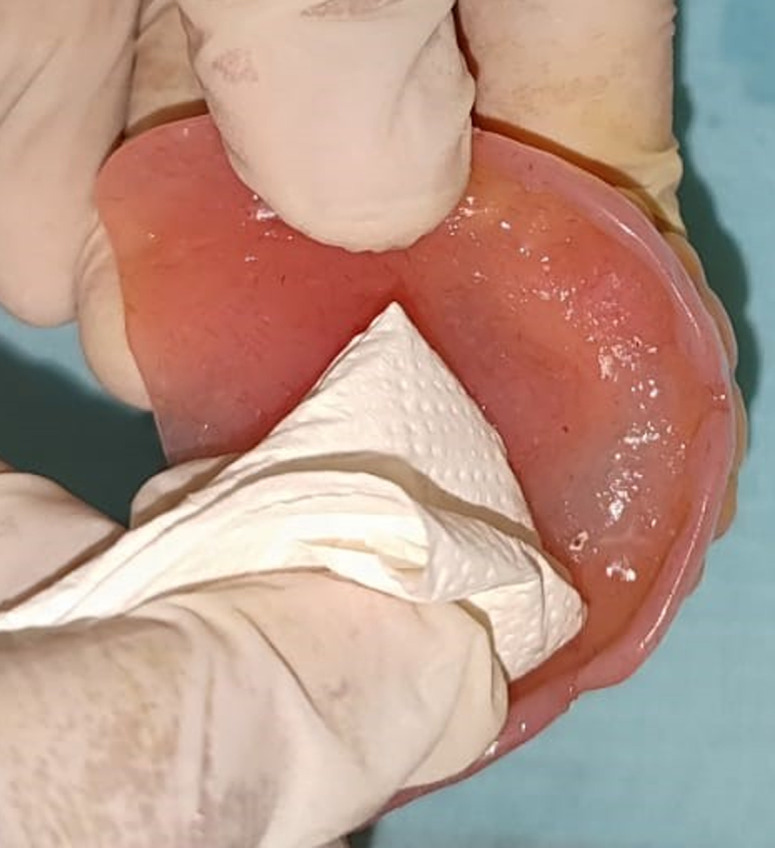
séchage de la prothèse

***Séchage ou humidification:*** la prothèse doit être sèche pour les crèmes et les bandes, mais légèrement humide pour les poudres, afin de faciliter l'adhésion (Figure 3) [5,6,11,13,15].

### Application de l'adhésif

**Figure 4:**
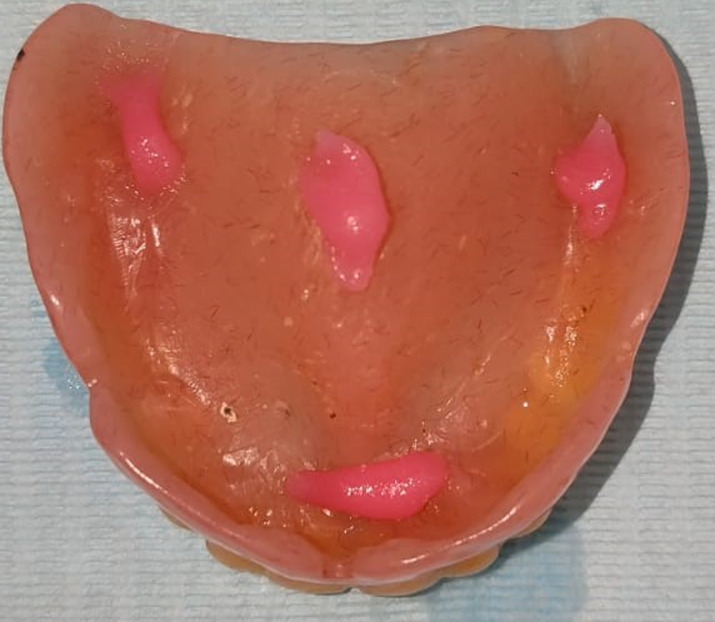
mise en place de la crème adhésive an niveau maxillaire

***Crèmes adhésives:*** pour la prothèse maxillaire, appliquer 3 ou 4 incréments de crème adhésive de la taille d'un petit pois, dans la crête antérieure, la zone médiane du palais et le bord postérieur, pour la prothèse mandibulaire, appliquer 3 incréments de crème adhésive de la taille d'un petit pois à plusieurs endroits de la crête (Figure 4).

**Figure 5:**
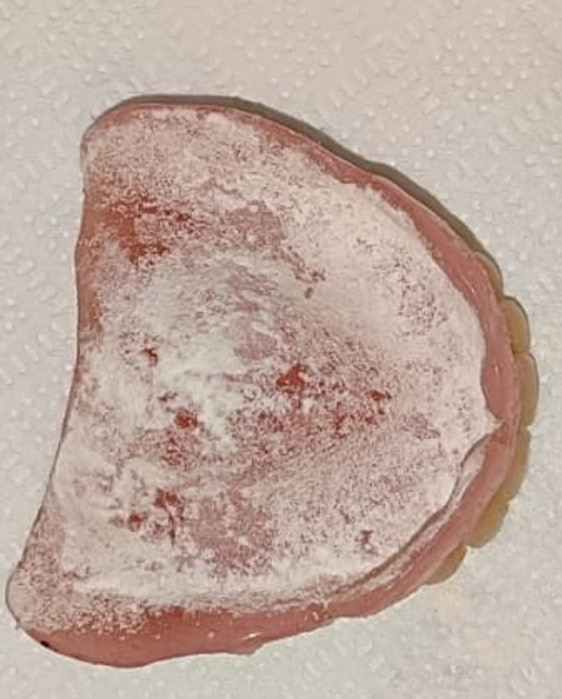
mise en place de la poudre adhésive sur prothèse humidifiée

***Poudres adhésives:*** appliquer une fine couche de poudre d'une manière uniforme sur la surface humide de la prothèse (Figure 5).

***Bandes adhésives:*** découper les bandes à la taille de la prothèse et les appliquer directement sur l'intrados de la prothèse, en évitant les bords pour prévenir les débordements.

***Adhésifs liquides:*** appliquer une fine couche de liquide adhésif sur la surface interne de la prothèse à l'aide de l'applicateur fourni [6,13,16].

**Figure 6:**
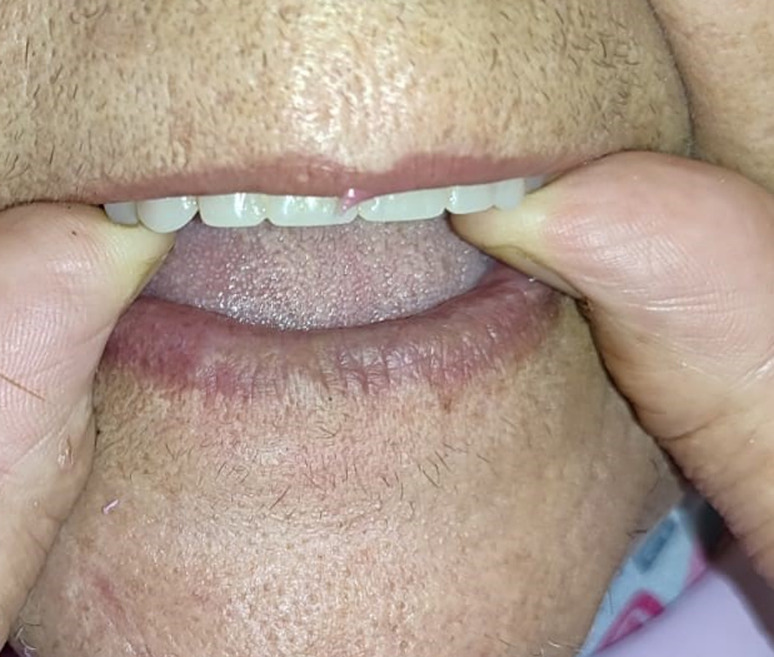
prothèse maintenue sous pression bidigitale

**Insertion de la prothèse:** une fois l'adhésif appliqué, la prothèse doit être positionnée de manière indépendante et maintenue sous une pression ferme et bidigitale (Figure 6) pendant quelques secondes afin de répartir uniformément l'adhésif et d'assurer une bonne adhésion à la muqueuse buccale [6].

**Figure 7:**
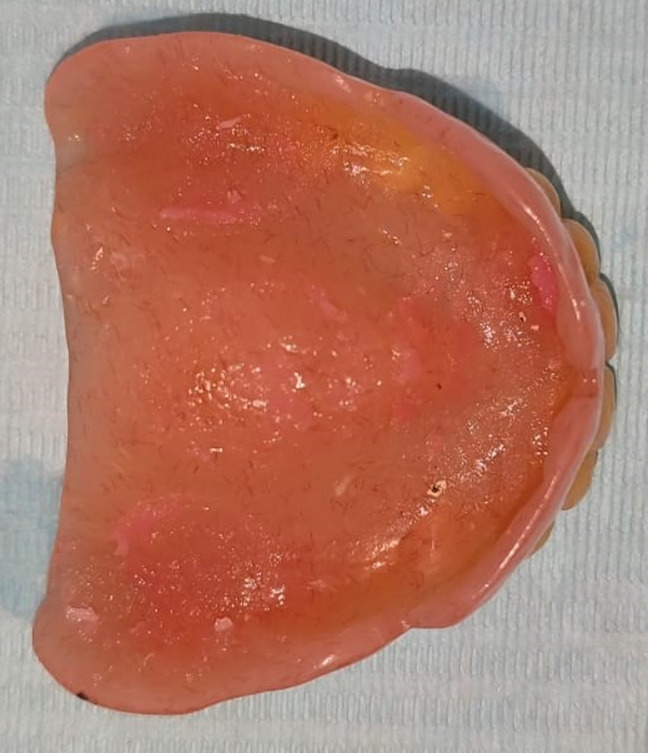
matériaux en place après des insertions

**Retrait et nettoyage:** il est conseillé de retirer l'adhésif des prothèses avec précaution pour éviter les blessures chaque jour (Figure 7). Il est ensuite nécessaire de brosser la cavité buccale afin d'éliminer les résidus d'adhésif sur les tissus mous. La prothèse doit être nettoyée à l'aide d'une brosse et d'un produit adapté, puis trempée dans un produit de nettoyage approprié [13,17,18].

## Discussion

Les adhésifs pour prothèses amovibles jouent un rôle essentiel dans l'amélioration de la rétention et de la stabilité des prothèses, surtout dans les cas de résorption osseuse sévère. Ils contribuent ainsi à l'optimisation de la biofonctionnalité des prothèses, offrant aux patients un meilleur confort lors de la mastication et de la parole. Cependant, il est essentiel que la prothèse soit correctement réalisée avant d'envisager l'utilisation des adhésifs. Ces derniers ne doivent pas compenser une mauvaise adaptation de la prothèse. Le choix de la forme d'adhésif en poudre, crème, gel ou bande doit être fait en fonction du contexte clinique et des besoins individuels du patient.

La poudre adhésive est recommandée pour les patients ayant une salivation normale, car elle interagit efficacement avec l'humidité buccale pour assurer une bonne adhésion. En revanche, la crème adhésive, offrant une adhérence plus forte, est plus adaptée aux patients présentant une résorption osseuse sévère. Les gels, plus confortables et faciles à appliquer, sont souvent préférés par les patients ayant une salivation réduite. Les bandes adhésives, quant à elles, sont très pratiques pour les patients ayant des limitations de dextérité. Une évaluation clinique approfondie, prenant en compte la résorption osseuse, la qualité de la salive et la capacité de manipulation du patient, est capitale pour déterminer la forme d'adhésif la plus appropriée [19-22].

L'utilisation d'adhésifs pour prothèses amovibles a été largement étudiée pour leurs effets sur la satisfaction des patients. Une revue systématique menée par Elabbasy *et al*. [23] a comparé l'efficacité des adhésifs chez les porteurs de prothèses complètes avec et sans utilisation d'adhésifs. Cette revue a conclu que l'utilisation d'adhésifs améliore significativement la rétention et la stabilité des prothèses, entraînant une satisfaction accrue chez les patients. Une autre revue systématique de Florêncio *et al*. [24] a montré que l'utilisation d'adhésifs améliore également les performances masticatoires chez les porteurs de prothèses, en augmentant l'efficacité de la mastication et en réduisant les déplacements prothétiques. Une étude multicentrique randomisée menée par To *et al*. [25] a également révélé que l'utilisation d'adhésifs chez les porteurs de prothèses complètes améliore significativement la qualité de vie liée à la santé bucco-dentaire, en augmentant la rétention et la stabilité des prothèses, contribuant ainsi à une meilleure fonctionnalité et à un plus grand confort. De plus, une autre étude d'Ereifej *et al*. [26] a montré que l'utilisation d'adhésifs améliore significativement la rétention, la stabilité et réduit l'accumulation de particules alimentaires. Une autre étude de Kurogi *et al*. [3] a révélé que l'utilisation d'adhésifs améliore non seulement la rétention des prothèses, mais aussi la force occlusale chez les patients porteurs de prothèses complètes, ce qui conduit à une amélioration des performances fonctionnelles lors de la mastication. De plus, une étude de Felton *et al*. [27] a révélé que l'utilisation d'adhésifs améliore les temps d'occlusion et de désocclusion, augmentant ainsi la stabilité des prothèses et le confort pour les patients.

En outre, une autre étude d'Adisman *et al*. [28] a montré que l'utilisation d'adhésifs prothétiques améliore non seulement la rétention et la stabilité, mais aussi les paramètres de mastication et les préférences alimentaires des patients, contribuant ainsi à une meilleure qualité de vie pendant la période d'intégration des prothèses. La biocompatibilité des adhésifs pour prothèses dentaires est un autre aspect fondamental à prendre en compte. L'étude de Papadiochou *et al*. [12] a révélé que, dans des conditions normales d'utilisation, les adhésifs modernes à base de polymères présentent une faible cytotoxicité, bien que certains produits puissent entraîner des irritations locales chez les patients présentant une sensibilité buccale accrue. Cependant, une mauvaise utilisation, un nettoyage insuffisant ou une utilisation prolongée peuvent favoriser l'apparition d'infections comme la stomatite prothétique. Une autre étude de Sampaio-Maia *et al*. [29] a montré que certains adhésifs peuvent favoriser la croissance de *Candida albicans*, un champignon responsable d'infections buccales fréquentes chez les porteurs de prothèses. Ces résultats soulignent l'importance d'une hygiène buccale stricte pour prévenir les infections fongiques potentielles.

Par ailleurs, certains adhésifs, notamment ceux contenant du zinc, ont soulevé des préoccupations concernant leur sécurité. L'étude de Felton *et al*. [30] a mis en lumière des cas de toxicité systémique liés à l'utilisation excessive d'adhésifs contenant du zinc, ce qui pouvait entraîner des déficiences en cuivre (hypocupremie) et des complications neurologiques. En réponse à ces préoccupations, les formules modernes ont éliminé le zinc, garantissant ainsi une meilleure sécurité. Une autre étude de Mekkawy *et al*. [31] a révélé que certains adhésifs et gels hydratants buccaux peuvent avoir des effets cytotoxiques modérés à élevés sur les cellules buccales, notamment les fibroblastes et kératinocytes humains. Cela souligne l'importance d'une évaluation attentive de la biocompatibilité des produits utilisés et de la durée de leur utilisation pour minimiser les risques pour les tissus buccaux.

En outre, une étude d'Ibraheem *et al*. [21] a démontré que l'application d'adhésifs pour prothèses pouvait réduire de manière significative la microdureté des matériaux de base en résine thermoplastique. Cette diminution de la dureté peut compromettre la durabilité et la fonctionnalité des prothèses complètes, ce qui suggère la nécessité d'une utilisation prudente des adhésifs et d'une surveillance régulière des prothèses.

## Conclusion

L'utilisation d'adhésifs pour prothèses amovibles, lorsqu'elle est bien adaptée aux besoins spécifiques des patients, améliore considérablement la satisfaction en termes de rétention, de stabilité et de confort lors de la mastication et de la parole. Les études montrent que ces adhésifs offrent des avantages significatifs en termes de fonctionnalité et de qualité de vie. Cependant, il est capital de maintenir une hygiène buccale rigoureuse et de veiller à une utilisation modérée des adhésifs afin de minimiser les risques d'irritation et d'infection. Les formulations modernes sans zinc ont permis de résoudre certaines préoccupations liées à la toxicité systémique, garantissant ainsi une utilisation plus sûre des adhésifs pour prothèses dentaires.

### Etat des connaissances sur le sujet

- Les adhésifs pour prothèses amovibles complètes sont utilisés depuis plusieurs décennies pour améliorer la rétention et la stabilité, en particulier dans les situations cliniques défavorables;
- Leur efficacité repose sur la formation d'une couche viscoélastique favorisant l'adhésion de la prothèse aux structures ostéo-muqueuses;
- Leur utilisation doit rester complémentaire et ne peut pas compenser une prothèse mal adaptée ou ne respectant pas les principes biomécaniques.

### Contribution de notre étude à la connaissance

- Cette revue propose une synthèse actualisée des propriétés biomécaniques, biologiques et psychologiques des adhésifs pour prothèses amovibles complètes;
- Elle précise les indications cliniques actuelles des adhésifs, en tenant compte de situations spécifiques telles que la résorption sévère, la xérostomie et les réhabilitations provisoires;
- Elle met en évidence l'importance d'un protocole d'utilisation rigoureux et souligne les avancées récentes en matière de sécurité, notamment l'abandon du zinc dans la majorité des formulations modernes.
